# Editorial: The spatial-temporal dynamics of host-pathogen interaction during inflammatory disease

**DOI:** 10.3389/fcimb.2023.1308419

**Published:** 2023-10-13

**Authors:** Donglei Sun, Xue Liu, Xiuli Yang

**Affiliations:** ^1^ Sheng Yushou Center of Cell Biology and Immunology, School of Life Sciences and Biotechnology, Shanghai Jiao Tong University, Shanghai, China; ^2^ Department of Pathogen Biology, International Cancer Center, Shenzhen University Medical School, Shenzhen, China; ^3^ Department of Veterinary Medicine, University of Maryland, College Park, MD, United States

**Keywords:** microscopy, host-pathogen interaction, imaging, fluorescence, intravital

The interaction between the host and invading pathogen determines the results of infection and disease outcome. Previous studies on host-pathogen interactions have largely relied on indirect methods to measure different parameters. In recent decades, with the development of novel imaging technology, witnessing the interplay between hosts and pathogens has become a new trend in the field. Novel imaging and labeling technologies have been development to allow imaging with higher spatial or temporal resolution, imaging on live animals with extended depth within tissue, or imaging on larger scale, such as a mouse organ or even a whole mouse. These technologies have enabled the characterization of host-pathogen interactions from various perspectives (**Figure 1**).

**Figure 1:**
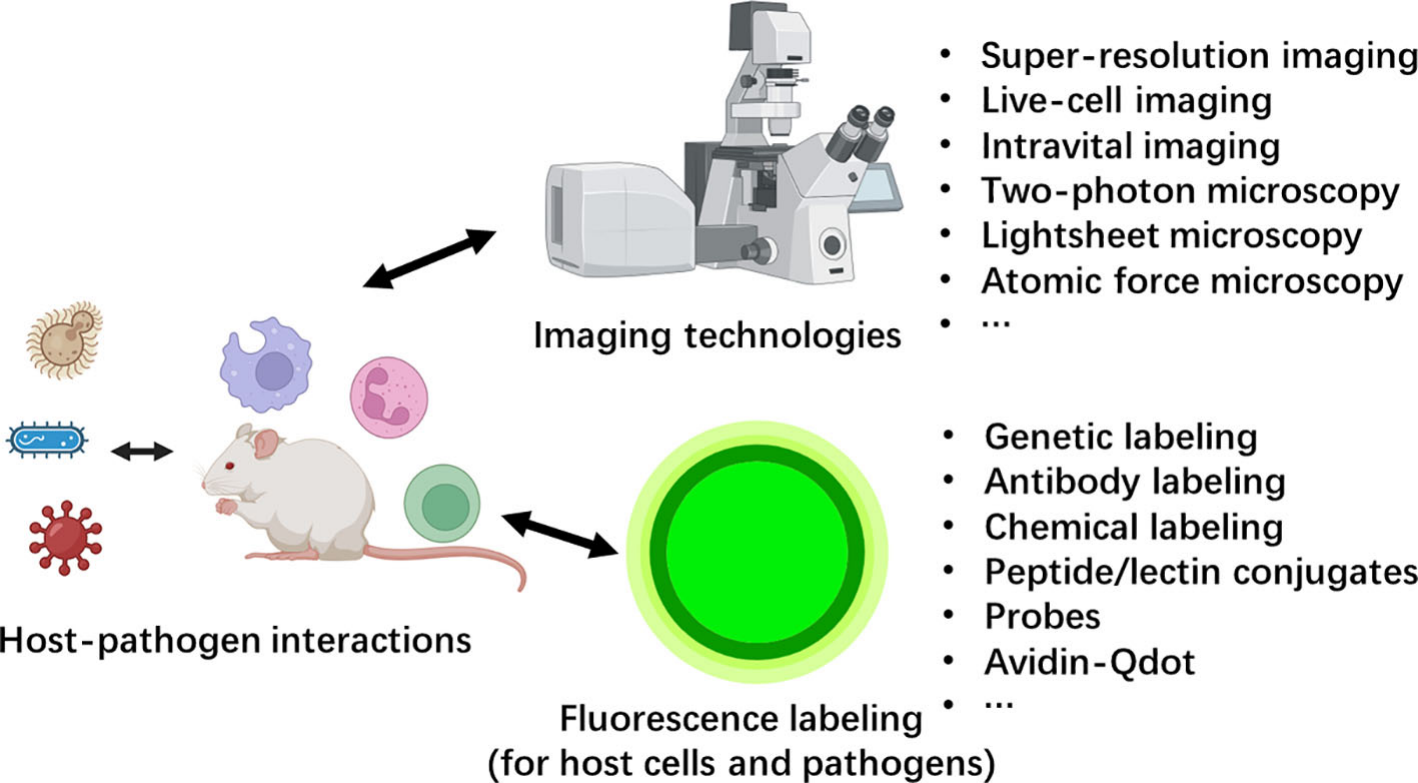
The advances in imaging technology and labeling strategies created a new era for host-pathogen interactions.

This Research Topic collected four recent studies that utilized different imaging techniques to visualize host-pathogen interactions. While the current topic doesn’t cover all advanced imaging technologies, it aims to set an example and encourages more future studies to utilizes various novel imaging techniques to unveil host-pathogen interactions.

Corliss et al. developed a plasmid-based reporter system, termed FlavER, to monitor flavivirus infection in real-time. This system utilized the viral protease to cleavage an ER-anchored fluorescent protein infection reporter fused to a nuclear localization signal (NLS). The release of NLS-reporter by the viral protease is translocated to the nuclei, which can be detected by fluorescence microscopy. By performing long term time-lapse imaging of living cells infected with dengue virus, they observed nuclear translocation of the reporter signal beginning approximately 8 hours post-infection, which continued to increase throughout the time course. Interestingly, they found that increased reporter signal translocation correlated with increased ER signal intensity. Collectively, this study presented a valuable tool for real-time monitoring flavivirus infection while studying the virus-dependent changes to the host cell ER at the same time.

Zhu et al. conducted a study on a common fungal pathogen Candida albicans. They labeled the fungi with a specific dye, Uvitex 2B, and used whole organ confocal imaging to visualize infected organs. Initially, they discovered that most fungi injected through the tail vein became trapped in capillary vessels. Furthermore, they examined the growth of fungal hyphae in different organs. Candida in the brain and kidney exhibited the most hyphal growth, indicating a lack of inhibition mechanisms at the early stage. In contrast, in other organs such as the lung, liver and spleen, far fewer fungi were able to grow hyphae. This differential growth of hyphae severed as a strongly indication of organ-specific immune responses. Further study using traditional methods like flow cytometry identified a dual wave of neutrophil recruitment at the early stage, which determined the disease outcome. This study underscored the application of confocal system for directly imaging animal organs when subjects are properly label with fluorescence.

Zeng et al. investigated the role of cytochrome b-c1 complex subunit 7 gene (QCR7) also in the fungal pathogen C. albicans. They first discovered and imaged the morphological changes of fungal colonies in knockout strains and visualized hyphal morphology using fluorescence microscope. These traditional imaging techniques provide insights into the virulence defect in the knockout strain. By fluorescently staining macrophages and neutrophils, they further revealed the reduced virulence was associated with diminished macrophage and neutrophil recruitment. Ultimately, this study concluded that the QCR7 gene of C. albicans promoted fungal colonization by enabling adaptation to multiple carbon source as well as regulation of filamentous growth and biofilm formation.

As more studies employ imaging techniques to investigate host-pathogen interactions, comprehensive reviews on this topic become increasingly necessary. Dendritic cell is the most prominent antigen presenting cell which plays a key role in the activation of adaptive immunity. Xiao and Xia have summarized recent advances on dendritic cell (DC) immunology during infection that utilized advanced imaging techniques. They began by providing an overview of the advantages and limitations of various major imaging techniques, such as intravital imaging, light-sheet microscopy, atomic force microscopy, and super-resolution microscopy, etc. Next, they introduced DC subsets, their sensing receptors, and signaling pathways. They further reviewed DC function in different organs and antigen processing pathways with emphasis on new discoveries made by advanced imaging. Finally, they reviewed current knowledge on mechanisms of DC migration and DC-T cell interaction during pathogen infection, and highlighted remarkable studies that have exploited imaging techniques.

In conclusion, this Research Topic comprises four exemplary studies that employ various imaging techniques to explore host-pathogen interactions. The application of advanced imaging technology not only allows researchers to study with unprecedented resolution and depth, but also enables examination of host-pathogen interactions within their real *in vivo* niches. However, it is important to acknowledge that not all advanced imaging techniques were covered in this Research Topic. Imaging techniques such as super resolution imaging, intravital imaging, atomic force microscopy, light-sheet microscopy were regrettably not included. Each of these techniques offers distinct advantages, We look forward to witness more studies that leverage these technologies in the future.

## Author contributions

DS: Writing – original draft, Writing – review & editing. XL: Writing – review & editing. XY: Writing – review & editing.

